# Prediction and classification of anxiety-related psychological scale and VR sickness based on autonomic physiological responses during VR treatment in patients with social anxiety disorder

**DOI:** 10.1192/j.eurpsy.2022.1479

**Published:** 2022-09-01

**Authors:** J.Y. Chun, H.-J. Kim, S. Lee, M. Kim, T. Cheong, C.-H. Cho

**Affiliations:** 1 Korea University, School Of Industrial And Management Engineering, Seoul, Korea, Republic of; 2 Chungnam National University Sejong Hospital, Psychiatry, Sejong, Korea, Republic of

**Keywords:** physiological response, social anxiety disorder, virtual reality, Anxiety

## Abstract

**Introduction:**

Social anxiety disorder (SAD) can accompany emotional symptoms as well as physical reactions. The assessment and real-time measurement of SAD is difficult in real-world.

**Objectives:**

This study aims to predict the severity of specific anxiety states and virtual reality (VR) sickness in SAD patients by a machine learning model based on only quantitative measuring of autonomic physiologic signals during VR therapy sessions.

**Methods:**

In total, 32 individuals with SAD symptoms were enrolled in VR participatory sessions. We assessed patients’ specific anxiety symptoms through Internalized Shame Scale (ISS) and Post-Event Rumination Scale (PERS), and VR sickness through Simulator Sickness Questionnaire (SSQ). Specific anxiety symptoms and VR sickness were divided into severe and non-severe states based on the total score of each scale by K-means clustering. Logistic regression, Random Forest, Naïve Bayes classifier, and Support Vector Machine were used based on the physiological signal data to predict the severity group in subdomains of ISS, PERS, and SSQ.

**Results:**

Prediction performance (F1 score) for the severity of the ISS mistake anxiety subdomain was higher than other scales with 0.8421. For VR sickness, prediction performance for the severity of the physical subdomain was higher than the non-physical subdomain with 0.7692.

**Conclusions:**

The study findings present that mistake anxiety and physical sickness could be predicted more accurately by only autonomic physiological signals, suggesting these features are probably associated with autonomic responses. Based on the present study results, we could provide the evidence for predicting the severity of specific anxiety or VR adverse effects only based on in-situ physiological signals.

**Disclosure:**

No significant relationships.

